# Weather conditions during hunting season affect the number of harvested roe deer (*Capreolus capreolus*)

**DOI:** 10.1002/ece3.7825

**Published:** 2021-06-27

**Authors:** Sophie Baur, Wibke Peters, Tobias Oettenheym, Annette Menzel

**Affiliations:** ^1^ Department of Life Science Systems ‐ Professorship of Ecoclimatology TUM School of Life Sciences Technical University of Munich Freising Germany; ^2^ Bavarian State Institute of Forestry (LWF) Freising Germany; ^3^ Institute for Advanced Study (IAS) Technical University of Munich Garching Germany

**Keywords:** behavior, climate change, game management, harvest success, ungulate

## Abstract

Due to human‐induced climate and landscape changes, distribution and abundance of many ungulate species have increased worldwide. Especially in areas where natural predators are absent, hunting is the essential management tool for regulating ungulate populations. Therefore, understanding the factors associated with harvest rates is the first step toward an adaptive management approach. Weather influences hunter and ungulate behavior and thus presumably harvest, but how and which meteorological parameters are linked to harvest numbers have rarely been evaluated. We used nearly 65,000 “sit and wait” and driven hunt harvests of roe deer (*Capreolus capreolus*) in Bavaria, Germany, and weather data from 2008 to 2017 to test for factors affecting roe deer harvests (i.e., temperature, rain hours, wind speed, sunshine duration, snow depth, workdays vs. weekends, month) using zero‐inflated negative binomial mixed‐effect models. Our results reveal that, besides workdays, high temperatures and prolonged rain resulted in fewer harvested animals, whereas sunshine duration in summer and snow height in snow‐rich areas partially favored harvests during sitting hunts in summer and winter, respectively. The influence of wind speed varied over the course of the year. In summer and autumn, wind speed commonly had a negative effect, positively affecting harvests in winter in some regions. Daily harvest numbers decreased during the summer and autumn hunting periods (May till mid‐October), while they increased during the winter period (mid‐October till mid‐January). Interestingly, harvest success during driven hunts, which are planned well in advance and therefore take place largely independent of weather conditions, was similarly affected by the weather. This result suggests that the inferred weather influence is not only due to the hunters' decisions but also due to deer behavior. Since many ungulate populations may further benefit from climate change, building an understanding of the relationship between hunting success and weather will aid adaptive ungulate management.

## Graphical abstract

Many ungulate populations are increasing worldwide due to favorable (climatic) conditions; without natural predators, hunting is the main management tool. We analyzed how weather (temperature, rain, wind, sunshine, snow) and other factors (weekdays, month) influence harvest numbers of roe deer over 10 years for two different hunting strategies. Our results reveal that, besides workdays, high temperatures and prolonged rain resulted in fewer harvested animals, whereas sunshine duration in summer and snow height in snow‐rich areas partially favored harvests during sitting hunts in summer and winter, respectively. The influence of wind speed varied over the course of the year. Importantly, the different strengths of weather factors on the number of harvested animals during sitting and driven hunts indicate weather influences on both hunters and roe deer.

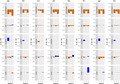

## 1 INTRODUCTION

Many ungulate species have expanded their distribution and increased in abundance throughout Europe and North America (Côté et al., 2004; Diekert et al., 2016; Milner et al., 2006). Factors that have facilitated these trends include increased availability of forage and suitable habitats due to phenological changes in response to climatic changes (e.g., milder and shorter winters) or agricultural and forest management, as well as reduced abundance or even absence of natural predators (Gortázar et al., 2000; Milner et al., 2006; Rickbeil et al., 2019). Like other ungulates, roe deer (*Capreolus capreolus*) primarily benefited from these changes. Roe deer are widespread throughout Europe (Putman et al., 2011) and are the most abundant ungulate in the federal state of Bavaria, Germany, with an increasing population trend over the last decades (Bayerisches Staatsministerium für Ernährung & Landwirtschaft und Forsten, 2019).

Due to increasingly favorable environments and the absence of natural predators, hunting is the main management tool for population regulation in many areas. State authorities commonly guide the management of game populations based on a hunting system, usually according to the age structure, sex ratio, and density. On the other hand, state authorities can influence hunting activities by restricting the hunting period, times of the day when hunting is allowed, hunting equipment, hunting methods, or the number of hunters per unit area (Mysterud et al., 2019; Stedman et al., 2004). In Bavaria, roe deer may be hunted between 5 and 9 months of the year depending on the respective sex and age class (relevant legal regulation: §19 AvBayJG), and hunters have the responsibility to fulfill the quotas set by the authorities. In contrast to many other countries, the comparatively long hunting period allows hunters to choose when to hunt to obtain their harvests freely.

The hunting success of natural predators depends, for example, on the individual (e.g., physical condition, experience), the target prey (e.g., physical condition, densities), and weather conditions (Mech et al., 2003). For hunters, the weather is also an important factor influencing their decision to go hunting, along with factors such as attitude, social norms, preference for target animals, knowledge, or time availability (Bhandari et al., 2006; Darimont & Child, 2014; Diekert et al., 2016; Kennan et al., 2008; Rivrud et al., 2014). Relationships between weather and hunting success have been shown for other (deer) species (Curtis, 1971; Fobes, 1945; Hansen et al., 1986; Leorna et al., 2020). For instance, for Virginia, USA, Curtis (1971) reported a negative relationship between white‐tailed deer (*Odocoileus virginianus*) seen and the mean daily temperature, but a positive one with total daily precipitation.

Upon such presumed weather effects, climate change could influence harvest numbers via altered prey activity patterns in response to weather and hunter decisions as well as environmental changes triggered by climate change (e.g., prolonged vegetation cover, favorable habitats resulting from natural disturbances). Here, we combined daily roe deer harvest and weather data covering ten years to test which weather factors affected the daily harvest rate of roe deer in seven regions across Bavaria. Such empirically based insights can be the first step toward an adaptive management approach.

**FIGURE 1:**
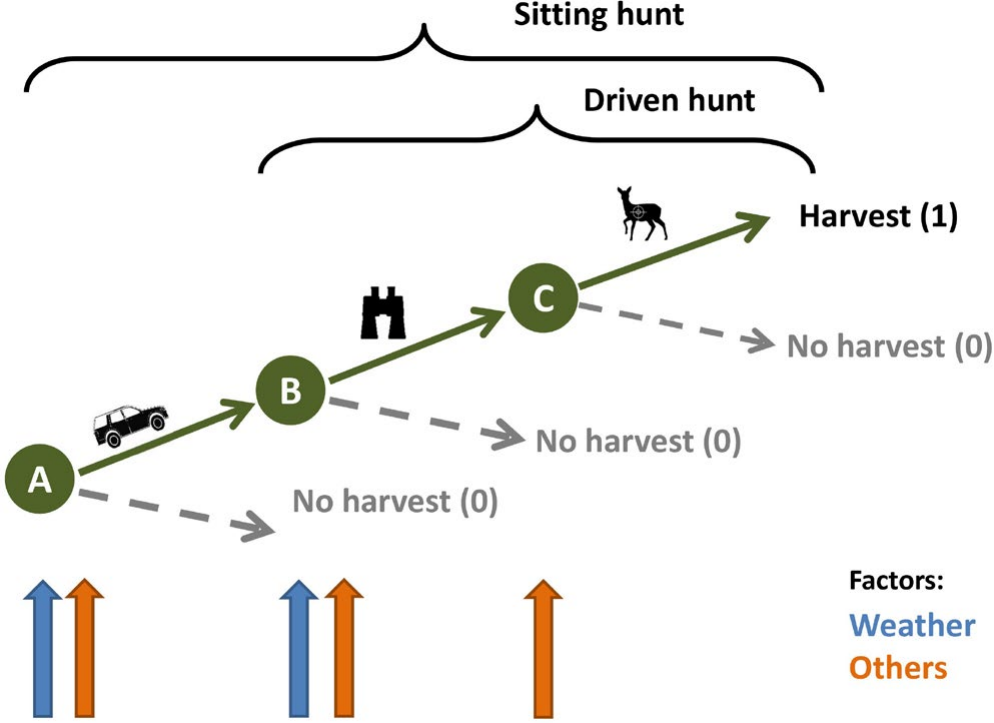
Scheme of hypothesized combined effects of weather and other factors on hunted roe deer numbers. (a) Decision of the hunter to go hunting; (b) sighting of roe deer; (c) successful hunt. Only if a, b, and c occur, a harvest date (=1) is recorded. We assume weather to influence both a and b, while other factors acting at all steps

We hypothesized that both hunters and roe deer alter their behavior in response to the weather. More specifically, the overall weather influence on successful hunts may include the combined probability (see scheme in Figure 1) of (a) a hunter going to the field (weather‐dependent decision to go hunting, especially true for “sit and wait” hunts, herein sitting hunts); (b) encountering roe deer during a hunt (weather‐dependent likelihood of sighting deer); and (c) actual hunting success during a hunt. While other parameters may also influence all three events, these other factors should have a stronger effect on b and c than on a (e.g., a: time available to a hunter for hunting, hunters' motivation; b: hunters' skills and experience in tracking animals, knowledge about the hunting area and of how deer may react to certain weather conditions, the influence of external disturbances; c: attitude, preferences, safety considerations, shooting skills).

Regarding weather, we expected a negative influence by wind and long‐lasting precipitation on harvest numbers as previously shown for roe deer, white‐tailed deer, or alpine chamois (*Rupicapra rupicapra*) (Brivio et al., 2016; Curtis, 1971; Hansen et al., 1986). With higher wind speeds and, therefore, more noise, it is more difficult for roe deer to identify potential threats, leading to increased vigilance and preferred use of safe habitats where deer are difficult to detect (Lone et al., 2014; Mysterud & Østbye, 1999). We also predicted lower harvest numbers with heavy or long‐lasting rain due to lower motivation of hunters to go hunting (Rivrud et al., 2014) and less visible deer because they use shelter and closed stands more frequently (König, 1987; Mysterud & Østbye, 1999). For temperature effects on harvest numbers of roe deer (Curtis, 1971; Progulske & Duerre, 1964), we predicted fewer harvests on hot summer days, associated with intense solar radiation, and cold winter days, because of less visibility due to the preferred use of cover for thermoregulation (Mysterud & Østbye, 1999). In winter, we expected a positive influence of the first snow due to a higher detection probability of deer by hunters (Zagata & Haugen, 1974), but an adverse effect with increasing snow depth as deer are likely to prefer forest stands with lower snow levels and thermal protection (Courbin et al., 2017). Due to a presumed higher motivation to go hunting on weekends (A), we expected a higher number of culled deer on Saturdays and Sundays than on workdays (Mysterud et al., 2019; Rivrud et al., 2014). Finally, to address the effect of weather on the hunters' decision to go hunting, an additional model was run exclusively for driven hunts. These hunts take place in winter, often on Fridays and Saturdays, and are planned far in advance (e.g., in summer), and concurrently, there should be no weather effects on the decision to participate in the hunt. Consequently, we expected more pronounced effects of weather‐related factors on the number of hunted roe deer for the sitting hunt models than for the driven hunt models.

## 2 METHODS

### 2.1 Roe deer hunting data

**FIGURE 2:**
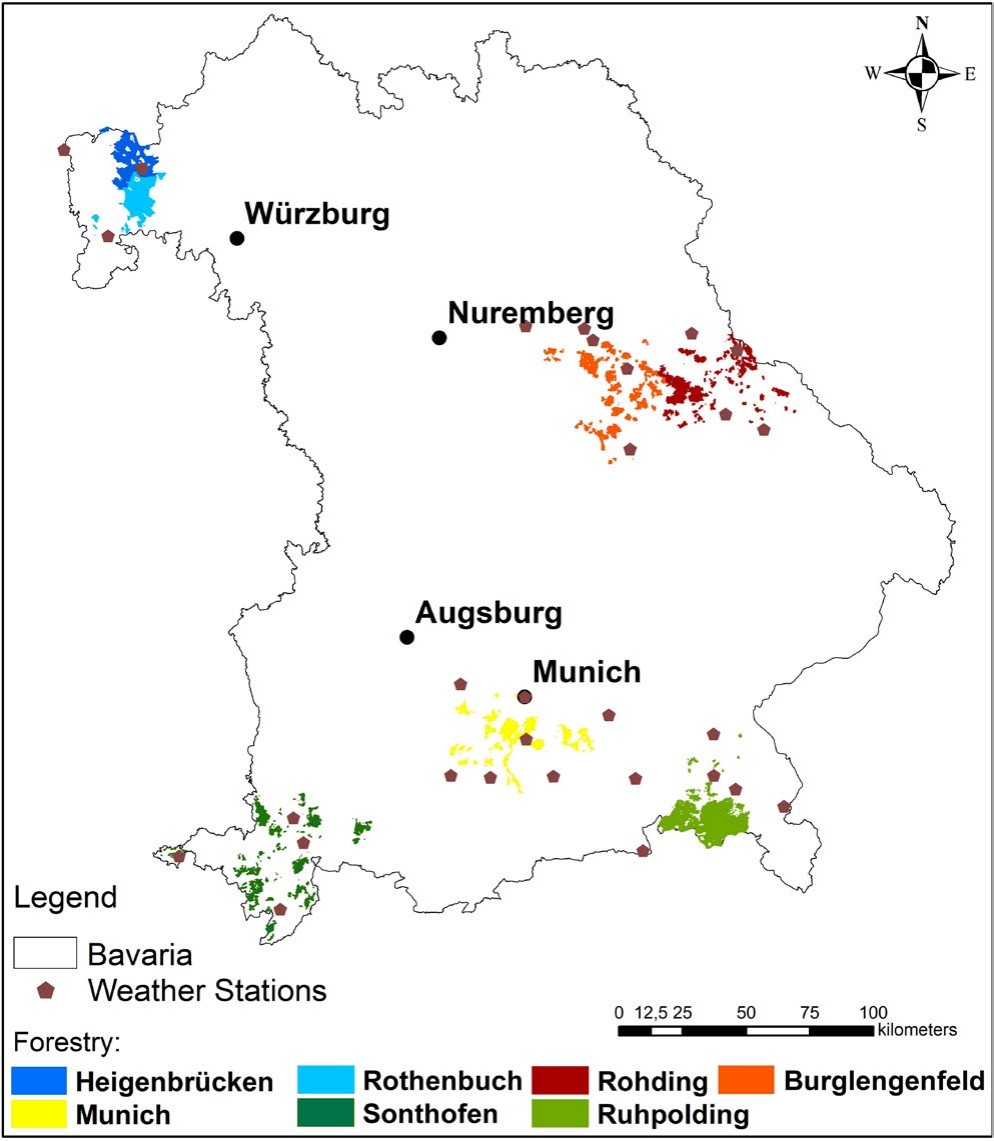
Location of the BaySF management units (regions) located in the German federal state of Bavaria and the location of the incorporated weather stations of the German Meteorological Service. Roe deer harvest data were collected in these regions between 2008 and 2017

Our study was conducted in Bavaria, the most southeastern federal state of Germany (Figure 2), located in the warm‐moderate climate zone with an average temperature of 7.8°C and 933 mm annual precipitation (period 1971–2000) (Bayerisches Landesamt für Umwelt, 2018). Roe deer is a native ungulate managed by binding 3‐year‐interval game harvest plans throughout Bavaria. The hunting authority sets harvest quota for each hunting ground according to the physical condition of harvested game and data on game browsing pressure (relevant legal regulation: Art. 32 BayJG). Depending on the level of browsing pressure, hunters may exceed or stay below the assigned quota (applicable legal statute: §16 Abs. 1 AVBayJG). Throughout Bavaria, the hunting season for bucks is between May 1 and October 15, for does and fawns between September 1 and January 15, and for female yearlings from May 1 to January 15 (§19 AvBayJG). Thus, we included the 9 months of May till January 15 in our analysis.

Hunting data were provided by the Bayerische Staatsforsten (BaySF), an institution under public law (AöR) responsible for managing approximately 808,000 ha state‐owned forests in Bavaria and conducting the wildlife management in about 823,000 ha. Ungulate management in 89% of the hunting area (including our study regions) is undertaken with the help of nearly 8,500 recreational hunters (thereof 4,300 paid hunting permit holders in 2017). Harvests by recreational hunters account for 75%, and 25% of the hunted deer are taken by 750 BaySF employees, including about 50 professional hunters. Game management strategies follow legal specifications, for example, ungulate densities should allow the regeneration of well‐adapted mixed and stable forests, preferably without fencing. Seventy‐six percent of all harvests are taken during single sitting hunts, 16% during driven hunts, 7% during collective sitting hunts, and 1% during guided hunts for guests (not applicable for roe deer) (Bayerische Staatsforsten AöR, 2011, 2017). For single sitting hunts, it can be assumed that most hunters are likely to hunt in areas near their residence or workplace, and hunts typically take a few hours, mostly during twilight hours (dusk, dawn). Thus, the time and place where the decision to go hunting is made is likely closely related to the time and place where hunt and harvest occur (Figure 1a–c). In contrast, for driven hunts, which commonly take place during the day in winter, the decision to go hunting by the individual hunter (Figure 1a) should be negligible since these hunts are planned and consequently signed up for well in advance. In remote alpine areas, a hunting session may require more time. Thus, taking a day off during the week for single hunting may be a common phenomenon to avoid disturbance due to those seeking relaxation on the weekends (personal communication, state hunting authority, and local heads of offices for Food, Agriculture and Forestry).

**FIGURE 3:**
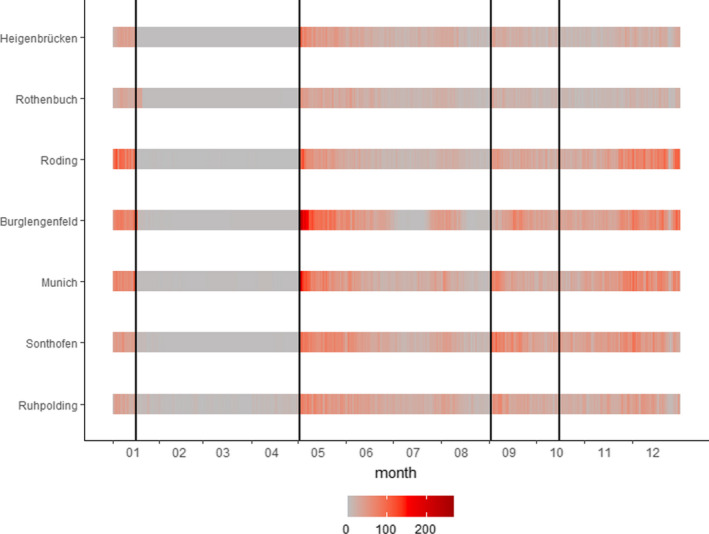
Sum of harvested roe deer (sitting hunt) between 2008 and 2017 per region and day of the year. The vertical lines indicate the hunting periods (summer—May 1 to August 31; autumn—September 1 till October 15; and winter—October 16 till January 15)

We analyzed daily roe deer harvest data from seven out of 41 BaySF management units (18.2% of the area) from May 1, 2008, to December 31, 2017 (Figure 3). Hereafter, we will call these seven forest management units "regions" for simplicity. Two regions are located in the northwest of Bavaria (Heigenbrücken, Rothenbuch) characterized by above‐average temperatures, the least number of snow days, and high percentages of deciduous forests (50% and 75%, respectively) (Table 1). Two regions are located in the conifer‐dominated region in the east (Roding, Burglengenfeld with 70% and 93% of conifers) characterized by low annual precipitation (778 mm and 699 mm, respectively). The two regions in the southern alpine area (Sonthofen, Ruhpolding) are dominated by mountain‐mixed forests (72% and 63% of conifers), and spruce‐stands under regeneration dominate one region in the southern center (Munich). The southern regions (Sonthofen, Ruhpolding) are characterized by the highest average rainfall and snow days and the lowest average annual temperatures (Bayerische Staatsforsten AöR, 2019).

**TABLE 1:** Overview of key environmental parameters per region

**A** Region	Temperature (°C)	Precipitation (mm)	Sunshine duration (h)	Snow days (days)
Heigenbrücken (a)	8.8 (7.4–9.9)	960 (757–1,135)	1602 (1,437–1,837)	42 (2–94)
Rothenbuch (b)	8.8 (7.4–9.8)	916 (718–1,089)	1,660 (1,452–1,856)	43 (2–98)
Burglengenfeld (c)	8.9 (7.1–9.9)	699 (543–798)	1,648 (1,459–1,856)	42 (15–102)
Roding (d)	8.3 (6.9–9.3)	778 (598–877)	1,660 (1,472–1,877)	51 (19–109)
Sonthofen (e)	6.1 (5,5–7.3)	1,904 (1,572–2,167)	1,725 (1,603–2,025)	125 (81–161)
Ruhpolding (f)	6.5 (5.0–7.2)	1,917 (1,582–2,128)	1,778 (1,580–2,049)	124 (75–157)
Munich (g)	8.7 (7.5–9.5)	1,057 (921–1,146)	1,777 (1,585–2,036)	51 (19–95)

**B** Region	Area (1,000 ha)	Species (deciduous/conifers)	Preferred species in regeneration (%) (2018)	Browsing damage (%) (2018)	Mean weight per piece (kg)	Percentage hunted in driven hunts
Heigenbrücken (a)	16.8	50/50	37	6.2	12.4	15.38
Rothenbuch (b)	17.0	75/25	48	5.9	11.7	6.09
Burglengenfeld (c)	21.2	7/93	57	12.1	11.7	10.14
Roding (d)	21.2	30/70	60	5.2	13.0	4.56
Sonthofen (e)	18.4	28/72	53	7.5	12.2	5.82
Ruhpolding (f)	34.5	37/63	70	14.1	12.4	4.72
Munich (g)	18.4	39/61	33	6.3	11.5	3.22

Note

A: Weather factors: 10‐year (2008–2017) means as well as annual minima and maxima (min‐max) for temperature, precipitation, sunshine hours, and snow days per year (based on data of the German Meteorological Service, DWD). B: Vegetation: tree species distribution in % (deciduous—beech, oak, maple); conifers—spruce, pine, fir, larch), percentage of species preferred by roe deer in the regeneration (fir, beech, oak, other hardwood deciduous), percentage of damaged trees in the regeneration by browsing damage on main shoots (data based on nature conservation concepts 2015/6 and results of browsing tract inventories 2018 of the Bayerische Staatsforsten AöR, 2015 & 2015).

Game management may slightly differ between regions due to climate, landscape, and species composition (Table 1b). But, we assume that the overall harvest strategy did not vary between regions except that professional hunters hunt in parts of the alpine regions Sonthofen and Ruhpolding in addition to recreational hunters. This also applies for parts of the Munich region (~10% of its area). The other regions have no additionally employed full‐time hunters.

Daily harvest data were available at the district level (mean size: 42.7 ha). Each region comprises 36 (Rothenbuch) to 90 (Ruhpolding) districts. Road‐kill and cases of natural mortality were excluded from the data set. In total, 60,269 harvests were included in the analysis for the sitting hunt models and 4,121 for driven hunt models. Driven hunts were mainly conducted between October and January in all regions and years by recreational and professional hunters. Harvest data were assigned to the nearest weather station (see below), and the data set was divided into days with harvests (number of harvested deer aggregated per climate station) and other days without harvests. Data on unsuccessful hunts were not available.

### 2.2 Weather data

For the study period, daily weather data of 97 climate stations were available from the German Meteorological Service (Deutscher Wetterdienst, 2020a, 2020c). We chose the nearest weather station for each district and used 29 stations (two to seven per region, see Figure 2). Wind speed was only available for ten and sunshine duration for 15 stations, so we used the closest station with this measurement alternatively. We also calculated rain hours per day as hours with precipitation ≥1 mm (Deutscher Wetterdienst, 2020b). The final set of explanatory weather factors comprised mean daily temperature (°C), sunshine duration (h/day), rain hours (h/day), and average wind speed per day (m/s) as well as daily snow depth (cm) for the winter months (October 15–January 15).

### 2.3 Statistical analyses

First, we tested for correlated explanatory variables at a Pearson rank correlation threshold of |*r*| ≥ 0.7. Only the variables mean precipitation and rain hours were highly correlated. Because the diurnal cycle of the amount of precipitation is more pronounced than the duration of precipitation in Germany (Ghada et al., 2019), we preferred rain hours/day over mean precipitation to be included in the models.

We modeled the number of shot roe deer per day for three specific periods of the hunting season separately to account for potential seasonal effects in hunting effort, deer behavior, environmental conditions, and, notably, the particular hunting seasons (see before). Specifically, we differentiated between "summer" from May 1 <>till August 31 on bucks and yearlings; "autumn" from September 1 till October 15 on bucks, yearlings, females, and fawns; and "winter" from October 16 till January 15 on females and fawns. The terms "summer," "autumn," and "winter" models thus relate to the hunting periods, not to the meteorological seasons.

To consider the excess zeros due to the assumed zero‐harvesting days in the models, we used a zero‐inflated negative binomial model (ZINB) with the glmmTMB package (Magnusson et al., 2019) in the statistical software R (R Core Team, 2019). ZINB was preferred over zero‐inflated Poisson (ZIP), generalized linear (GLM), or negative binominal (NB) models as indicated by a significant Vuong test (Vuong, 1989, *p* < .05). In general, glmmTMB has been shown by Brooks et al. (2017) to be more flexible for estimating those models via maximum likelihood estimation and faster than packages that use Markov chain Monte Carlo sampling.

To account for potential interannual variations, we included year as a random effect. The factors workday versus weekend and month were implemented to test for possible variation during the week (workday = Monday till Friday, weekend = Saturday, Sunday) and during the hunting periods (reference categories are May in summer, September in autumn, and October in winter models). Workday and month combinations may also account for the factor time availability (gun light hours not overlapping with standard working hours, see Figure 1a).

We calculated 21 separate sitting hunt models for the seven regions and three hunting periods to reveal possible differences between the management units. Furthermore, for all regions and periods of the hunting season, an overall model including region as a random effect was calculated. Driven hunts were limited to the winter hunting season, and we did not have enough data to fit robust models for the individual regions. Therefore, only one model for all studied regions in Bavaria was built and compared to the respective overall sitting hunt model in terms of (significant) variables and effect magnitudes.

We applied an ordered‐backward stepwise selection approach using the buildmer package (Voeten, 2019) based on AIC to identify the most parsimonious model. For all models, we calculated the relative risk (RR) for an increase of one unit per variable [RR = exponential (estimated parameter)] (RR > 1: higher risk of being shot/RR < 1: lower risk of being shot/RR = 1: average risk of being shot). The variable effects were compared in terms of inclusion in the model and the magnitude and direction of RR deviation from 1.

To compare the depicted effect sizes across weather variables, we additionally assessed ΔRR. For temperature, sunshine duration, and wind speed, we calculated the respective ΔRR by as many units as corresponding to the distance between the upper and lower quantiles, for example, comparing RR on a cold (25% quantile) and a warm (75% quantile) day. For rain hours and snow depth, we calculated ΔRR as the median of values >0, that is, the effective influence of rainy and snowy days compared to days without rain or snow.

## 3 RESULTS

The total number of harvested roe deer ranged from 4,285 to 11,731 per region and from 3,434 (August) to 13,170 (May) per month over the 10 years analyzed. Hunters shot most roe deer on May 1 when the hunting season started, while numbers were lower in the following months of the hunting season (Figure 3). In summary, 26,048 culls were included in the (sitting hunt) summer model (16,983 bucks, 9,065 female yearlings), 9,835 in the autumn (1,255 bucks, 3,223 females, 5,357 fawns), and 24,386 in the winter model (10,228 females, 14,158 fawns).

### 3.1 Weather effects on harvest numbers

The relative risk (RR) of roe deer harvest was considerably driven by weather and calendar variables, summarized in Table 2 for the seven regions and the overall model in the three hunting periods. Among the meteorological variables, temperature, wind speed, and snow depth were always selected (24 cases, but of course, only 8 cases for snow depth in the winter models), and rain hours in almost all cases (23 of 24). Sunshine was the least frequently selected variable (6 of 24 cases, but all in summer models). However, the temperature was significant in almost all cases (23 of 24 occurrences), while for the other variables, only 50% to 66% of their occurrences were significant (snow depth 4/8, rain hours 15/23, wind speed 15/24, sunshine duration 4/6). Thus, temperature can be considered the most important factor for RR. Uniformly, higher temperatures always (i.e., in all hunting seasons and regions) resulted in lower numbers of harvested roe deer (RR between 0.90 and 0.98), and this temperature effect was always significant except in the Rothenbuch autumn model.

**TABLE 2:** Relative risks for roe deer being harvested per region and the overall model

Region	Variable	Summer	Autumn	Winter
Heigenbrücken	Temperature	0.91 (1.009)***	0.95 (1.013)***	0.90 (1.010)***
	Rain hours	0.97 (1.010)**	0.98 (1.013).	1.01 (1.008)
	Sunshine	1.03 (1.008)***		
	Wind speed	0.95 (1.031).	0.93 (1.041).	1.02 (1.028)
	Snow depth			0.99 (1.009)
	Workday	0.79 (1.058)***	0.78 (1.088)**	
	Month (6–10–11)	0.66 (1.078)***	0.57 (1.107)***	0.86 (1.127)
	Month (7‐na−12)	0.66 (1.090)***		0.86 (1.136)
	Month (8‐na−01)	0.56 (1.090)***		1.38 (1.150)***
Rothenbuch	Temperature	0.94 (1.009)***	0.98 (1.013)	0.95 (1.009)***
	Rain hours	0.98 (1.009)*		1.01 (1.007)
	Sunshine	1.01 (1.007)		
	Wind speed	0.96 (1.028)	0.92 (1.041)*	0.99 (1.027)
	Snow depth			0.98 (1.009)*
	Workday	0.92 (1.055)		
	Month (6–10–11)	0.85 (1.069)*	0.65 (1.115)***	0.99 (1.124)
	Month (7–na−12)	0.67 (1.083)***		1.40 (1.134)
	Month (8–na−01)	0.56 (1.086)***		1.59 (1.144)***
Burglengenfeld	Temperature	0.98 (1.007)*	0.97 (1.009)**	0.95 (1.106)***
	Rain hours	0.98 (1.007)***	0.99 (1.009)	1.00 (1.005)
	Sunshine	0.99 (1.006)*		
	Wind speed	0.92 (1.030)***	1.01 (1.039)	1.01 (1.019)
	Snow depth			1.00 (1.004)
	Workday	0.87 (1.043)***	0.84 (1.064)**	0.85 (1.043)***
	Month (6–10–11)	0.48 (1.054)***	0.74 (1.074)***	1.00 (1.069)
	Month (7‐na−12)	0.21 (1.078)***		1.09 (1.076)
	Month (8‐na−01)	0.25 (1.071)***		1.17 (1.086).
Roding	Temperature	0.95 (1.026)***	0.96 (1.009)***	0.93 (1.005)***
	Rain hours	0.97 (1.007)***	0.96 (1.009)***	0.99 (1.005)**
	Sunshine			
	Wind speed	0.95 (1.026)*	1.07 (1.028)*	1.04 (1.015)**
	Snow depth			0.99 (1.004).
	Workday	0.92 (1.048).	0.83 (1.064)**	0.90 (1.041)*
	Month (6–10–11)	0.50 (1.063)***	0.64 (1.078)***	1.15 (1.074).
	Month (7‐na−12)	0.38 (1.077)***		1.40 (1.087)***
	Month (8‐na−01)	0.40 (1.073)***		1.68 (1.085)***
Sonthofen	Temperature	0.94 (1.006)***	0.96 (1.009)***	0.93 (1.006)***
	Rain hours	0.97 (1.005)***	0.98 (1.007)**	0.99 (1.004)**
	Sunshine			
	Wind speed	0.77 (1.042)***	0.91 (1.062)	1.18 (1.030)***
	Snow depth			0.99 (1.003)***
	Workday			
	Month (6–10–11)	0.79 (1.056)***	0.57 (1.074)***	0.99 (1.072)
	Month (7‐na−12)	0.53 (1.068)***		0.82 (1.081)*
	Month (8‐na−01)	0.56 (1.067)***		0.70 (1.095)***
Ruhpolding	Temperature	0.93 (1.007)***	0.97 (1.010)**	0.94 (1.008)***
	Rain hours	0.97 (1.006)***	0.99 (1.007)*	0.98 (1.006)***
	Sunshine	1.01 (1.006).		
	Wind speed	0.95 (1.022)*	1.00 (1.030)	1.02 (1.018)
	Snow depth			1.04 (1.004)***
	Workday			
	Month (6–10–11)	0.92 (1.057)	0.61 (1.082)***	0.87 (1.094)
	Month (7‐na−12)	0.72 (1.066)***		0.68 (1.104)***
	Month (8‐na−01)	0.61 (1.067)***		0.62 (1.126)***
Munich	Temperature	0.93 (1.007)***	0.98 (1.010)*	0.93 (1.006)***
	Rain hours	0.99 (1.006)	1.00 (1.008)	1.00 (1.005)
	Sunshine	1.02 (1.007)**		
	Wind speed	0.82 (1.024)***	0.92 (1.032)**	0.95 (1.013)***
	Snow depth			1.01 (1.005)*
	Workday	0.88 (1.047)**	0.85 (1.075)*	0.75 (1.047)***
	Month (6–10–11)	0.54 (1.063)***	0.61 (1.088)***	1.53 (1.081)***
	Month (7‐na−12)	0.48 (1.073)***		1.49 (1.087)***
	Month (8‐na−01)	0.37 (1.075)***		1.62 (1,098)***
Overall Model	Temperature	0.94 (1.003)***	0.96 (1.004)***	0.93 (1.003)***
	Rain hours	0.98 (1.003)***	0.98 (1.003)***	0.99 (1.002)***
	Sunshine	1.01 (1.003)***		
	Wind speed	0.90 (1.010)***	0.98 (1.013).	1.01 (1.007)
	Snow depth			1.00 (1.002)
	Workday	0.92 (1.018)***	0.91 (1.027)***	0.89 (1.020)***
	Month (6–10–11)	0.65 (1.023)***	0.63 (1.031)***	0.74 (1.100)*
	Month (7‐na−12)	0.48 (1.028)***		0.82 (1.069).
	Month (8‐na−01)	0.44 (1.028)***		0.85 (1.039)***

Note

Equally to relative risk transformed standard errors in parentheses.

Significance: significant at 0.1, * significant at 0.05; ** significant at 0.01; *** significant at 0.001.

Relative risks are given for an increase of one unit per selected variable from the parsimonious negative binominal zero‐inflated models per region and hunting period (summer = May, June, July, August; autumn = September, October 15, winter = October 16, November, December, January 15). Month effects are compared to May (summer), September (autumn), and October (winter hunting period).

With few exceptions in the winter models (Heigenbrücken RR 1.01, Rothenbuch RR 1.01, Burglengenfeld RR 1.00, Munich RR 1.00, all nonsignificant), the number of rain hours had a negative effect on the harvest success, although its influence was comparatively small (RR 0.96‐0.99, 15 of 21 significant).

The effect of wind speed varied throughout the year. In summer, the relative risk of harvest was consistently significantly reduced by higher wind speed, and the effects ranged from RR 0.77 (Sonthofen) to RR 0.95 (Heigenbrücken, Ruhpolding, and Roding) (only Rothenbuch RR 0.96 not significant). In contrast, in autumn and winter, the effect of wind speed was mixed in sign and significance. Four of eight autumn models indicated significant negative effects of wind speed (Heigenbrücken RR 0.93, Rothenbuch RR 0.92, Munich RR 0.92, and the overall model RR 0.98), and a significant positive relationship was observed in Roding (RR 1.07). Six of eight winter models showed a positive influence of wind speed (RR between 1.01 and 1.18, significant in Rohding and Sonthofen); however, Munich's effect was significantly negative (RR 0.95).

The effect of sunshine duration was restricted to the summer models and was associated with a higher number of hunted roe deer five times, although this effect was relatively small (RR up to 1.03 at Heigenbrücken, Rothenbuch, Ruhpolding, Munich, overall model), and once negative (Burglengenfeld R 0.99).

In the winter models, snow depth had a consistent but small negative influence in Heigenbrücken, Rothenbuch, Roding, and Sonthofen (RR 0.98‐0.99), no effect in Burglengenfeld and the overall model, and only in Ruhpolding (RR 1.04) and Munich (RR 1.01) did higher snow depths lead to higher harvest numbers.

### 3.2 Calendar effects on harvest numbers

Interestingly, the factor month was selected for all hunting period models (all regions and the overall model), indicating within‐hunting period variations in relative risk. In summer, hunting success was significantly lower in June, July, and August compared to May (only in Ruhpolding June was not significant), often decreasing from June throughout July to August (median RR 0.66, 0.51, and 0.50, respectively). The corresponding single RR ranged from 0.21 (Burglengenfeld July) to 0.92 (Ruhpolding June). In autumn, October yielded significantly fewer harvested roe deer in all regions compared to September (RR between 0.57 and 0.74, median 0.62). In winter in the two alpine regions, Ruhpolding and Sonthofen, RR decreased steadily from November to January compared to October (minimum RR 0.62 Ruhpolding January). In contrast, RR was positive and increased continuously from November to January in Roding, Munich, Burglengenfeld, and Rothenbuch (except November) (maximum RR 1.68 Roding, January). In Heigenbrücken, January showed the strongest positive influence compared to October (RR 1.38).

Workday had a negative effect on the number of harvested deer in 15 of 24 cases, with all but one case being significant, that is, the risk for roe deer to be shot was higher on weekends than on workdays (RR 0.92‐0.75, median RR 0.87). There was no workday effect depicted for the alpine regions Sonthofen and Ruhpolding as well as for Heigenbrücken in winter and for Rothenbuch in autumn and winter.

### 3.3 Driven hunts in winter

We compared the RR of roe deer to be shot in winter for driven hunts (Table 3) to the respective overall sitting hunt model (Table 2). Weather effects were comparable but even more pronounced in the driven hunt model: significantly negative for both temperature (RR 0.92 and 0.93, respectively) and rain hours (RR 0.97 vs. 0.99), and significantly positive for wind speed (RR 1.12 vs. 1.01). Snow depth significantly negatively affected the number of harvested deer during driven hunts (RR 0.92), whereas it had no effect on the overall sitting hunt model for the winter hunting period (RR 1.00). The sunshine duration had a nonsignificant negative effect only in the driven hunt model. Workdays had a much higher effect (RR 0.44) on harvest risk during driven hunts than sitting hunts (RR 0.89). Compared to October, risk rates in November and January drastically increased (RR 2.64 and 2.05, respectively) in the driven hunt model. Thus, in contrast to our prediction, the weather influence did not decrease for the preplanned and organized driven hunts, for which the weather influence on hunters should be negligible (see Figure 1a).

**TABLE 3:** Relative risks for driven hunts in the winter hunting period

variable	RR
Temperature	0.92 (1.013)***
Rain hours	0.97 (1.012)*
Sunshine	0.97 (1.020)
Wind speed	1.12 (1.035)***
Snow depth	0.92 (1.013)***
Workday	0.44 (1.110)***
Month 11	2.64 (1.161)***
Month 12	1.69 (1.182)**
Month 01	2.05 (1.218)***

Note

Equally to relative risk transformed standard errors in parentheses.

Significance: significant * at 0.05; ** at 0.01; and *** significant at 0.001 level.

The overall model relative risk calculations (RR) are given for an increase of one unit per selected variable in the most parsimonious negative binominal zero‐inflated model. Month effects compared to October.

### 3.4 Effect size of weather variables

**FIGURE 4:**
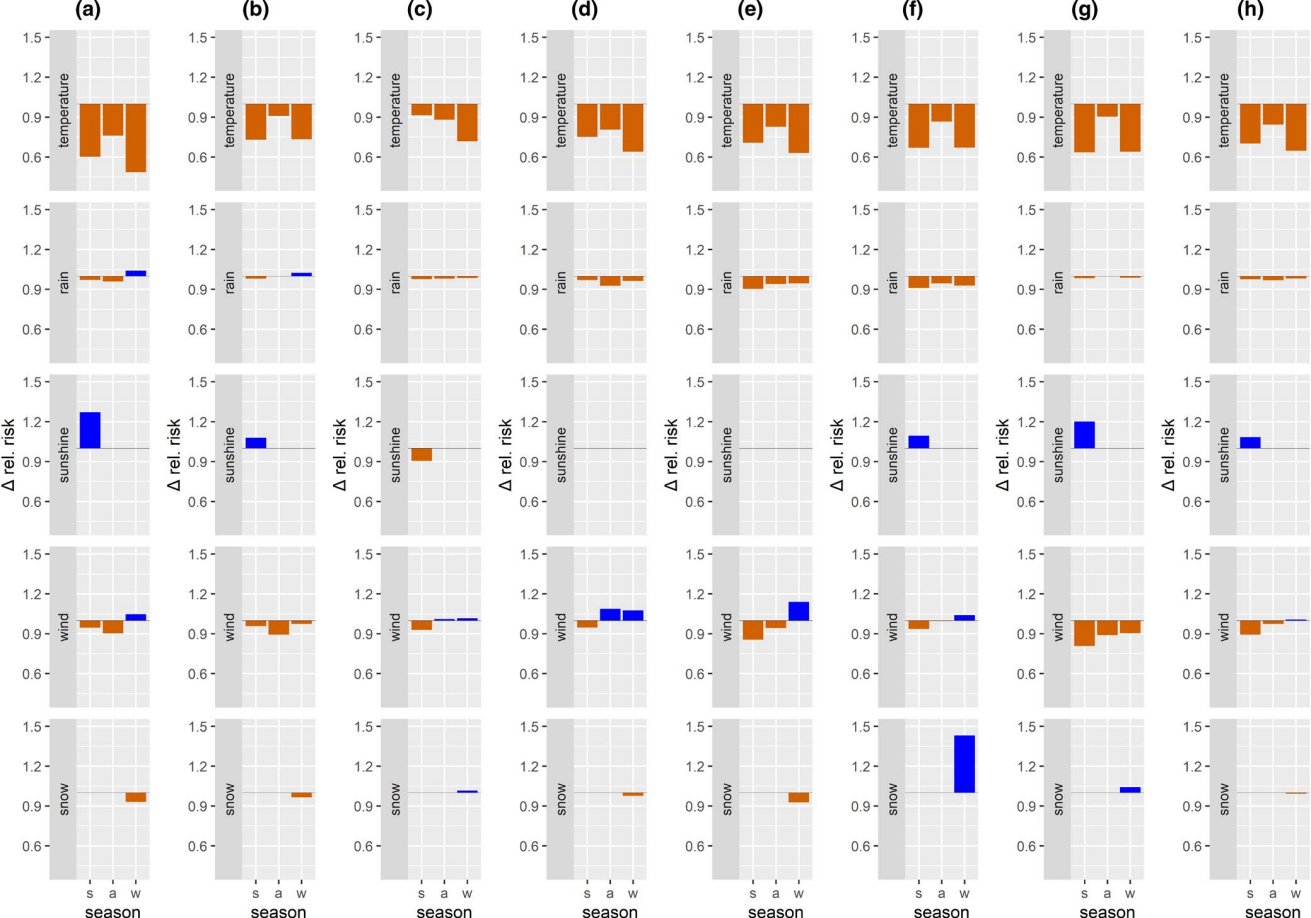
Relative risks for quantiles and median values (ΔRR) compare the influence of different meteorological variables on roe deer harvest risk. Relative risk calculations are for the distance between upper and lower quartiles (temperature, sunshine, wind) and median values for rain and snow >0. Results of the parsimonious sitting hunt negative binominal zero‐inflated models are given per region ((a) Heigenbrücken, (b) Rothenbuch, (c) Burglengenfeld, (d) Roding, (e) Sonthofen, (f) Ruhpolding, (g) Munich, (h) overall model) and hunting period (summer—May 1 to August 31; autumn—September 1 till October 15; and winter—October 16 till January 15)

The ΔRR values, which indicate the effect size, for example, for temperature, the difference between a medium warm and a medium cold day (75%– 25% quantile), allow a comparison of different meteorological variables within each model (Figure 4 and Table S1). Temperature exhibited the largest deviations of ΔRR from 1 and therefore had the strongest effect in reducing the RR (i.e., ΔRR 0.50 Heigenbrücken in winter). ΔRR for temperature was always stronger in summer and winter than in autumn models (except Burglengenfeld). The positive effect of sunshine duration was smaller in effect size than for temperature in five summer models (Heigenbrücken, Rothenbuch, Ruhpolding, Munich, and the overall model), with ΔRR values between 1.27 (Heigenbrücken) and 1.08 (overall model). In Burglengenfeld, on the other hand, the reducing effect size of sunshine was equal to that of temperature (ΔRR 0.91). The strongest positive RR effect of snow in winter (RR 1.04 Ruhpolding; Table 2) translated into a ΔRR of 1.41, indicating a 1.41 higher risk on days with than without snow cover. In contrast, the ΔRR of rain hours and wind speed within each model indicated a considerably smaller influence than temperature.

## 4 DISCUSSION

The number of harvested roe deer in seven regions of Bavaria was linked to meteorological parameters and could thus also be influenced by climate change in the long run. The traditionally anchored assumptions of inhibiting influences of rain and wind on harvest success could be confirmed (or partially in the case of wind), whereas the results on favoring influences by sun and snow were less clear. Sun and wind were seasonally restricted to summer and winter, respectively, and may require more intensive investigations on larger data sets. Higher temperatures were most importantly associated with pronounced lower harvest numbers across all hunting periods and regions, both for sitting and for driven hunt models. In Bavaria's largely recreational hunter‐dependent hunting system, daily harvests were greater on weekends than on workdays and exhibited changes as the hunting periods progressed. Following a general discussion on the effect pathway of the weather influence, we will discuss the effects of each weather variable as well as calendar effects to derive management options from these findings.

### 4.1 Self‐fulfilling prophecy or independent weather effect?

Our data represent the combined effect of hunter effort, skill, behavior, and external circumstances such as weather, although the modeling approach could only take total weather effects and calendar variables into account. Workday–weekend differences clearly indicate a hunter availability issue, much like that reported by Mysterud et al. (2019) for recreational hunters in Norway. This latter study also lists other nonmeteorological factors, such as hunter skills and attitude, potential disturbance by visitors, safety considerations regarding the shot, which we could not disentangle in our study.

Weather could theoretically act at three nodes (see Figure 1), namely (a) the hunter's decision to hunt, (b) deer sighting probability, and (c) the successful hunt. Since other authors have shown a high correlation between indices of seen deer per hunter and harvested number per hunter (Simard et al., 2012), we assume that the weather effect on the last step (c) should be small. Consequently, the two first nodes (a, b) are likely stronger influenced by weather parameters, either directly (actual weather) or indirectly (hunter experience and decisions regarding weather). For example, the deer sighting probability (b) depends on both hunter and deer behavior. In adverse weather conditions, deer avoid open areas and preferentially use cover (Mysterud & Østbye, 1999). Thus, movement rates are low, and animals are less visible to hunters (Lone et al., 2014). At the same time, also the motivation to go hunting may be lower during unfavorable weather conditions. Thus, roe deer harvests may be related to both hunter and roe deer responses to weather. Further analyses to separate the effects of weather on hunting success would require additional survey data, such as the number of days hunted or successful versus nonsuccessful hunts, data that were unfortunately not available for our study.

However, we took the following approach also to test whether all weather effects are just some sort of self‐fulfilling prophecy by hunters, such as "hunting deer in high winds is not going to be successful." We have compared driven hunt to sitting hunt models during the winter hunting season. Driven hunts are special events that are planned well in advance in summer; thus, without considering the weather, hunters commit their participation, and dog handlers are recruited in time. Therefore, there is good reason to believe that these hunts occur with no (or almost no) influence of weather on the decision to go hunting. Consequently, for driven hunts, we expected that weather parameters should have less impact on harvest numbers. However, we found a similar effect of temperature and a slightly stronger influence of rain. On the other hand, a larger positive influence of high wind speeds was detected, probably because beaters and dogs move roe deer in driven hunts, and therefore, their movements are much less affected by wind. Thus, contrary to our hypothesis, the weather influence was comparable between sitting hunts and driven hunts, except for wind speeds. Consequently, our comparison of sitting and driven hunt models suggests that weather effects are not solely attributable to the hunter's decision to hunt (Figure 1a) but are rather influenced by both hunter and roe deer behavior (b and c).

### 4.2 Temperature effects

Among the meteorological variables, the temperature had the strongest overall effect on harvest rates. It was always (i.e., for all regions and hunting periods) included in the most parsimonious models, uniformly fewer roe deer were harvested at higher temperatures, and in relative terms, the temperature had the strongest absolute influence. It is known from the literature that spring temperatures positively influence roe deer activity (Pagon et al., 2013). Compared to the May–July period, harvest numbers in August were generally lower, most likely due to a behavioral change during the rut (Krop‐Benesch et al., 2013; Picardi et al., 2019), but also likely due to lower hunting effort during summer vacation. While the temperature was the main factor explaining the white‐tailed deer detectability during spotlight surveys in summer (Progulske & Duerre, 1964), the effect of high temperatures on harvest rates is ambiguous in the literature. For example, declines in hunting success have been reported in some studies of white‐tailed deer or Dall sheep (*Ovis dalli dalli*) when temperatures are higher than average (Curtis, 1971; Leorna et al., 2020), but other studies have also reported contrary results (Fobes, 1945). In general, people prefer outdoor activities at higher temperatures up to ~27°C (Prettenthaler et al., 2015). If recreational behavior can be assumed for hunters, a higher number of hunters should be active at higher temperatures, leading, for example, to more deer seen (Curtis, 1971). However, perhaps there is a trade‐off between hunting and pursuing alternative recreational activities when temperatures are ideal. The temperature influence on hunting success varied with season and land cover type in other studies (Rivrud et al., 2014), but in our study, the influence of temperature was uniform throughout the year. In winter, hunters typically assume higher chances of harvesting game at lower temperatures because bait sites are visited more frequently (Ossi et al., 2017; Ossi et al., 2020). Consequently, hunters may increase their effort during these periods.

### 4.3 Rain and wind effects

Rain hours often decreased harvest success in the summer and autumn hunting periods and partly in winter, although their influence was comparatively small and significant only in ~2/3 of the cases. This effect was noticeable in the southern alpine areas with high rainfall for all seasons. In the comparably drier parts of northern Bavaria, rain even had a positive effect on roe deer harvest numbers in the winter model. The influence of precipitation is controversially discussed in the literature, ranging from no effect on the likelihood to shoot a red deer (*Cervus elaphus*) (Diekert et al., 2016) to a negative effect on daily harvest of white‐tailed deer (Hansen et al., 1986) and Dall sheep (Leorna et al., 2020), or even increased harvest rates with more frequent precipitation days (Fobes, 1945). The latter result was explained by better and calmer hunting conditions when the forest floor was moist. Rain only slightly influenced white‐tailed deer movements (Webb et al., 2010) or had no effect (Beier & McCullough, 1990). Additionally, precipitation may also influence hunter behavior. In general, fewer outdoor activities are conducted during rainy periods (Spinney & Millward, 2011), and in a survey of hunters, precipitation was cited as one of the worst conditions for hunting (Curtis, 1971).

Like temperature, the wind speed was selected in all models and was significant in half of them, but its effect direction and magnitude varied. Our results showed a negative influence on the likelihood of roe deer being harvested in summer and (partially) in autumn. First, strong winds can create risky conditions for being outdoors. In general, wind makes it difficult for hunters to encounter deer undetected. The winter models suggested a higher than average risk for roe deer being shot with wind speed in all but two regions (Rothenbuch, Munich), although it is particularly difficult to explain the strong positive influence of high wind speed during winter in Sonthofen and Roding (autumn, winter). Possibly topography and land cover with smaller forest areas in Sonthofen play a role because high wind speeds cause deer to avoid open (alpine) pastures and seek shelter in forests. Another explanation is that professional hunters in these regions may cope better with less suitable conditions and still hunt more effectively than recreational hunters, who dominate in the other regions.

### 4.4 Sunshine in summer and snow in winter

Sunshine duration only positively influenced the number of harvests five times in the summer model, once a negative and twice no influence. In addition, these effects were rather small. According to the literature, white‐tailed deer showed higher activity rates during cloud‐free and cold days and selected open areas to benefit from higher solar radiation (thermoregulation) (Beier & McCullough, 1990). Our study cannot separate whether the influence of sunshine is related to the potential thermoregulation that roe deer may seek or hunter preferences. However, we suppose that sunshine hours rather have a stronger effect on the hunters because closed forests dominate most regions in this study, and cover for thermoregulation is provided nearly everywhere.

The effect of snow depth in the winter hunting season varied in magnitude and direction among the regions. In 50% of the models' snow slightly decreased harvest rates. In the presence of snow, higher hunting success is assumed due to better tracking possibilities (Fobes, 1945) and easier and more reliable identification of animals due to the higher contrasts with the surrounding (Mysterud et al., 2019). In contrast, there is evidence that deer decrease activity rates even at low snow depths due to locomotive constraints or lower food availability (Beier & McCullough, 1990; Gaudry et al., 2015). Even in our most southern alpine study regions with more snowfall, the influence of snow was only strong in Ruhpolding but not in Sonthofen. We assume that snow cover is most variable at the onset of winter, whereas, during the peak of winter and in alpine areas, high snow cover generally inhibits hunting activities.

### 4.5 Calendar effects

Workday versus weekend undeniably made a difference because, in 15 of 18 models for nonalpine regions, harvest rates were greatly reduced on workdays. This most likely reflects different frequencies in human hunting activity (Ciuti et al., 2012) instead of weather effects. For example, the workday was one of the strongest predictors of hunting effort and success on red deer in Norway, with higher culling rates on weekends (Mysterud et al., 2019; Rivrud et al., 2014), and also elk (*Cervus canadensis*) in Idaho, USA, were more frequently killed on weekends (Gratson & Whitman, 2000). This reducing influence of workdays was found for regions without professional hunters (Heigenbrücken, Rothenbuch, Roding, Burglengenfeld, and Munich for 90% of the area), where recreational hunting permit holders are predominantly active in addition to other BaySF employees. Interestingly, the variable workday was not selected for the two alpine regions (Sonthofen and Ruhpolding). This discrepancy could be because touristic activities hinder hunting in the Alps on weekends and change animal behavior, known as the “weekend effect” (Nix et al., 2018). Thus, professional hunters might concentrate their activities on workdays, and even recreational hunters may prefer to take one day off during the week to reach their remote hunting areas. Yet, in general, hunters who hunt during workdays are considered more effective than casual ones (Rivrud et al., 2014), an observation that we cannot evaluate due to a lack of data on hunter effort (e.g., number of hours spent hunting).

We also observed temporal patterns within hunting periods, namely decreasing harvests over time in summer and autumn and increasing during the winter hunting period, except in the snow‐rich southern regions. It is reasonable to assume that hunter effort decreases as the hunting season progresses on specific target animals (e.g., the hunting season on bucks and yearlings starting on May 1 yielded the maximum daily harvest) and may again increase when the annual hunting season closes in January to meet quotas (Diekert et al., 2016). A human‐induced pattern of shooting more reindeer early in the season and on weekends was also reported by Mysterud et al. (2019) for Norway. However, an indirect effect of hunting on habitat selection by roe deer is possible as they may alter their use of space with the start of the hunting season (Bonnot et al., 2013) to avoid risky habitats (Padié et al., 2015).

### 4.6 Outlook on future management

Looking at the observed climatic changes in Bavaria during the last seven decades (1951–2019), it can be concluded that our findings on the effects of weather on hunting success may complicate game management, at least for roe deer, in the future. Mean temperatures significantly increased across all seasons (by 2.1, 2.1, 2.4, and 1.2°C for winter, spring, summer, and autumn, respectively), and precipitation decreased significantly in summer but increased in the other seasons. The frost‐free period has increased by 27 days over the last seven decades (Bayerisches Staatsministerium für Umwelt und Verbraucherschutz, 2020). Moreover, ongoing climate change will likely lead to more forest disturbances, such as bark beetle infestations, potentially further increasing the availability of forage and suitable habitats due to rejuvenating stands, and milder winters may reduce natural mortality in the future. Consequently, roe deer populations will likely benefit from these climate‐driven environmental changes.

In contrast, wildlife management and hunting, in particular, could become more complex. Warmer springs have already advanced the start of the green wave, that is, leaf unfolding has shifted from May to April (Menzel et al., 2020), making it more challenging to detect game when the hunting season begins (May 1 for roe deer in Bavaria). Less snow in winter and/or a shift in the snow period (Fontrodona Bach et al., 2018) toward the end and after the hunting season may reduce hunting success in winter. Our study supported these concerns and added another previously unknown factor: high temperatures are associated with a reduced number of hunted deer in all seasons. We assume that experienced and knowledgeable hunters who are aware of these relationships may capitalize on this weather dependency in animal behavior and increase their likelihood of success. Thus, the influence of weather on roe deer behavior could translate into similar weather‐responsive but preadaptive hunter behavior.

## 5 CONCLUSION

A next step to consolidate and improve our findings would be to include new data on hunter behavior and unsuccessful hunts, which were not available for our study. Since we did not have information on hunting effort per unit time, the number of hunters or deer sighted, it was impossible to account for these confounding factors, and our results show the combined effect of all possible influences. Since large, systematic data sets on successful and unsuccessful hunts are not currently available, experimental approaches could help disentangle the effects between animals, humans, and weather. Similarly, our analysis could not account for other confounding factors, such as (also weather‐driven) human or other disturbances on wildlife and hunting activities, for example, recreational activities. Although we are aware of this shortcoming, we were interested in understanding the net weather effect on the harvests.

We fitted models for each hunting period, assuming constant hunter effort across them. However, harvests in individual seasons are not independent. A deer can only be killed once. Thus, high success at the beginning of the hunting season lowers game densities at the end and vice versa. Especially at the start of the respective hunting season, careless and less experienced animals may be shot and/or are easier to hunt. Moreover, strictly mathematically, the maximum hunting quotas would have to be considered as censored data. However, we believe it is reasonable to assume that hunting effort is intensified at the end of winter (as suggested by our models) if it is foreseeable that the annual quota will not be met (Diekert et al., 2016).

Increasing ungulate densities and concurrent climatic changes can modify the landscape in which both wildlife and hunters operate and live. For example, because ungulate densities can alter plant species richness and forest stand composition and regeneration, that is, factors that will also contribute to the resilience of forests to climatic changes, research‐driven adaptation is needed to adjust ungulate management. Study results such as those presented here can contribute to our understanding of wildlife management under changing climatic conditions. Specifically, in the hunting system we investigated, this could be implemented through regionally adapted spatio‐temporal hunting strategies. For example, an interval hunting system with varying intensity of hunting pressure to create spatio‐temporally structured risk landscapes (Norum et al., 2015) would be one possibility. Hunting could be stopped completely during the more unsuccessful summer months to compensate for more intensified hunting pressure when success rates are higher. Also, the end of the hunting season for bucks in October could be extended. Hunters should be encouraged to conduct hunts, preferably in suitable weather conditions, and on workdays rather than concentrate their hunting activities on weekends. Research on how climatic changes will affect roe deer populations and especially spatio‐temporal patterns of habitat use at multiple scales will provide further information for adaptive management strategies. Overall, ungulate management strategies need to be adapted to local environmental conditions, ungulate densities, and the specific hunting system.

## CONFLICT OF INTEREST

None declared.

## AUTHOR CONTRIBUTIONS

**Sophie Baur:** Conceptualization (equal); formal analysis (lead); methodology (equal); validation (equal); visualization (equal); writing–original draft (lead); writing–review and editing (equal). **Wibke Peters:** Conceptualization (supporting); methodology (supporting); supervision (equal); writing–review and editing (equal). **Tobias Oettenheym:** Formal analysis (supporting); methodology (supporting); visualization (supporting). **Annette Menzel:** Conceptualization (supporting); data curation (equal); funding acquisition (supporting); methodology (supporting); project administration (equal); supervision (lead); writing‐review and editing (equal).

## Supporting information

Table S1

## Data Availability

Climate data are freely accessible from the German Meteorological Service data server: http://opendata.dwd.de/climate_environment/CDC/observations_germany/climate/. The hunting data set is not freely downloadable for reasons of traceability and privacy regulations of the hunters. The data owner is an institution under public law (BaySF AöR) that assures long‐term archiving of data and provisioning of data, following a specific law of public information on environmental data.
