# Supplementary material for: Weather conditions during hunting season affect the number of harvested roe deer (Capreolus capreolus)
Source: Ecol Evol. 2021 Jun 27;11(15):10178–91. doi: 10.1002/ece3.7825 (PMC8328461; doi:10.1002/ece3.7825)
Supplement: Supplementary file 1 — Table S1 [file ECE3-11-10178-s001.docx]

**Weather conditions during hunting season affects the number of harvested roe deer (Capreolus capreolus)**

Sophie Baur^1*^, Wibke Peters^2^, Tobias Ottenheym^1^, Annette Menzel^1,3^

^1^ Department of Ecology and Ecosystem Management, Professorship of Ecoclimatology, Technical University of Munich, Freising, Germany

^2^ Bavarian State Institute of Forestry (LWF), Freising, Germany.

^3^ Institute of Advanced Study, Technical University of Munich, Garching, Germany

* Corresponding author

Email: sophie.baur@tum.de

**Supporting Information**

Attachment Table S1. **Relative risk for quantiles and median values (ΔRR) comparing the effect sizes among variables on roe deer risk of being harvested.** Relative risk calculations are for the differences between upper and lower quartiles (temperature, sunshine, wind) and median values for rain and snow >0. Results of the parsimonious sitting hunt negative binominal zero-inflated models per region and hunting season are given.

| region | variable | summer | autumn | winter |
| --- | --- | --- | --- | --- |
| Heigenbrücken | temperature | 0.60 | 0.76 | 0.50 |
|  | rain hours | 0.97 | 0.96 | 1.05 |
|  | sunshine | 1.27 |  |  |
|  | wind speed | 0.95 | 0.90 | 1.02 |
|  | snow depth |  |  | 0.90 |
|  |  |  |  |  |
| Rothenbuch | temperature | 0.73 | 0.91 | 0.73 |
|  | rain hours | 0.98 |  | 1.02 |
|  | sunshine | 1.08 |  |  |
|  | wind speed | 0.96 | 0.89 | 0.97 |
|  | snow depth |  |  | 0.96 |
|  |  |  |  |  |
| Burglengenfeld | temperature | 0.91 | 0.88 | 0.71 |
|  | rain hours | 0.98 | 0.98 | 0.99 |
|  | sunshine | 0.91 |  |  |
|  | wind speed | 0.97 | 1.01 | 1.01 |
|  | snow depth |  |  | 1.01 |
|  |  |  |  |  |
| Roding | temperature | 0.75 | 0.81 | 0.65 |
|  | rain hours | 0.97 | 0.93 | 0.97 |
|  | sunshine |  | 1.00 |  |
|  | wind speed | 0.95 | 1.09 | 1.06 |
|  | snow depth |  |  | 0.97 |
|  |  |  |  |  |
| Sonthofen | temperature | 0.71 | 0.83 | 0.63 |
|  | rain hours | 0.90 | 0.94 | 0.94 |
|  | sunshine |  |  |  |
|  | wind speed | 0.86 | 0.94 | 1.14 |
|  | snow depth |  |  | 0.92 |
|  |  |  |  |  |
| Ruhpolding | temperature | 0.67 | 0.87 | 0.67 |
|  | rain hours | 0.91 | 0.94 | 0.93 |
|  | sunshine | 1.09 |  |  |
|  | wind speed | 0.94 | 1.00 | 1.03 |
|  | snow depth |  |  | 1.41 |
|  |  |  |  |  |
| Munich | temperature | 0.64 | 0.90 | 0.65 |
|  | rain hours | 0.98 | 1.00 | 0.99 |
|  | sunshine | 1.20 |  |  |
|  | wind speed | 0.81 | 0.89 | 0.90 |
|  | snow depth |  |  | 1.04 |
|  |  |  |  |  |
| Overall Model | temperature | 0.70 | 0.84 | 0.65 |
|  | rain hours | 0.98 | 0.97 | 0.98 |
|  | sunshine | 1.08 |  |  |
|  | wind speed | 0.89 | 0.97 | 1.01 |
|  | snow depth |  |  | 0.99 |
